# The Role of Purine Metabolism-Related Genes PPAT and IMPDH1 in the Carcinogenesis of Intrahepatic Cholangiocarcinoma Based on Metabonomic and Bioinformatic Analyses

**DOI:** 10.1155/2023/5141836

**Published:** 2023-01-20

**Authors:** Chang-Jun Liu, Zhong-Zhi Ma, Wei-Zhi Gong, Xian-Hai Mao, Hao-Quan Wen, Xiao-Hui Wang

**Affiliations:** Department of Hepatobiliary Surgery, Hunan Provincial People's Hospital (The First Affiliated Hospital of Hunan Normal University), Changsha 410005, Hunan Province, China

## Abstract

In this study, we investigated the role of tumor microenvironment and serum differential metabolites in intrahepatic cholangiocarcinoma (ICC) carcinogenesis, providing new evidence for ICC treatment. Serum samples from healthy individuals and ICC patients were collected for metabolomic analysis. The purine metabolites such as inosine, guanosine, hypoxanthine, and xanthine were increased in patient serum. TCGA database samples were collected, and the correlation between purine metabolism-related genes and ICC clinical features was analyzed using R language to obtain the differential genes including PPAT, PFAS, ATIC, and IMPDH2. High PPAT expression was associated with poor ICC prognosis. A PPAT silencing model in HCCC-9810 cells was constructed. The cell phenotype was examined by qRT-PCR, CCK-8, transwell, and flow cytometry, showing a decrease in IMPDH1 expression, colony and invasive cells numbers, and an increase in apoptosis. Guanosine reversed IMPDH1 expression in HCCC-9810 cells, promoting the secretion of inflammatory factors IL-6, IL-8, OPN, VEGF, and VCAM-1 and intensifying epithelial-mesenchymal transition (EMT) progression in the cells. In nude mice, the IMPDH1 inhibitory drug MMF inhibited tumor growth and reduced the expression of tumor stem cell characteristic markers CD133 and SOX2. Guanosine accelerated the malignant progression of ICC inhibition of purine metabolism-related genes, PPAT and IMPDH2, suppressed the malignant phenotype in HCCC-9810 cells, and inhibited tumor growth.

## 1. Introduction

Intrahepatic cholangiocarcinoma (ICC) is a high-mortality malignancy with a steadily increasing incidence worldwide [1]. ICC is usually asymptomatic in its early stages and is usually diagnosed in an advanced stage when symptoms appear and diffused disease is detected, which limits the current potential treatment options based on surgery or liver transplantation [2]. In the clinical setting, primary biliary diseases are clinically distinct but show some similar features, such as chronic cholestasis, idiopathic disease, and high risk of malignant transformation [3]. Therefore, the differential metabolic phenotypes observed in the sera of patients with malignant and benign cholestatic diseases may provide clinical value and increase available diagnostic modalities [4]. The role of differential metabolites in the carcinogenic process of ICC and their regulatory mechanisms needs to be investigated urgently.

Metabolomics is one of the latest histological techniques that uses robust analytical techniques to screen biological samples for low-molecular weight metabolites that are closely associated with functional alterations in the body [5]. Metabolomics allows us to better understand the pathological processes and substance metabolic pathways by analyzing and validating specific biomarkers of a disease. Even small metabolite changes can help detect early pathological changes with better sensitivity than traditional diagnostic methods [6]. Given the potential of metabolomic analysis for biomarker discovery, our aim was to study the metabolites of interest for analysis and to presuppose that lipids, bile acids, high-energy metabolites, and amino acids could be potential biomarkers for biliary cancers. Purine metabolism is associated with carcinogenic effects and has been reported to be altered in the urinary biochemical profiles of patients with hepatocellular carcinoma [7]. Our pre-experimental results revealed that the purine metabolite products such as inosine, guanosine, hypoxanthine, and xanthine were elevated in ICC.

Guanosine monophosphate (GMP) plays a crucial role in energy metabolism, cell signaling, and cell reproduction [8]. Despite the important functions of the genes involved in the purine ab initio synthesis pathway, little is known about their regulation and in vivo expression patterns [9]. Purine metabolite synthesis is dependent on phosphoribosyl pyrophosphate amidotransferase (PPAT) [10]. It has also been reported that genes with oncogenic effects form a metabolically distinct subpopulation with abundant purines, which is dependent on inosine monophosphate dehydrogenase (IMPDH), an enzyme for guanosine triphosphate (GTP) biosynthesis [11]. In this subgroup, induction of IMPDH1 expression and dependence was necessary and sufficient, and IMPDH1 inhibitors were able to inhibit tumor growth in xenograft and in situ genetically engineered mouse models [12]. Screening of purine metabolism-related genes from TCGA database revealed that PPAT and IMPDH1 were associated with ICC survival. Therefore, the next step in studying ICC involves the regulation of PPAT and IMPDH.

PPAT transfers the *γ*-nitrogen of glutamine to 5-phosphoribosyl pyrophosphate (PRPP), a key rate-limiting reaction in purine biosynthesis [13, 14]. Indeed, PPAT expression is increased in lung adenocarcinoma and correlates with patient prognosis [15]. PPAT has been less studied in ICC, and the link between PPAT and IMPDH1 remains unclear. Enhanced IMPDH1 activity could facilitate the provision of highly depleted GTP nucleotides in highly proliferating stem cells [16]. Lymphocytes undergo intense proliferation in the presence of high guanine nucleotide depletion in response to an antigenic challenge. During the proliferation of antigen-activated T cells, IMPDH1 polymerization forms cytoplasmic structures [17]. IMPDH1 cell cytosolic assembly makes the enzyme more resistant to feedback inhibition of guanosine-di/trisphosphate (GDP/GTP) transitions, thus promoting the accumulation of guanine nucleotide pools [18]. This finding implies that IMPDH1 can potentially influence the immune microenvironment. We hypothesized that PPAT and IMPDH1 affect the proliferation, apoptosis, migration, and invasive functions of ICC.

However, the results of previous studies have shown significant differences in purine metabolites between normal patients and patients with ICC. Nevertheless, whether guanosine, a purine metabolite, can regulate ICC function and the immune microenvironment to inhibit tumor progression is unclear. The present study clarified the high levels of guanosine in patients with ICC. Expression of PPAT and IMPDH1 was higher in ICC. Exogenous supplementation with mycophenolic acid (MPA), a drug that inhibits IMPDH1, suppresses the malignant phenotype of ICC, inhibits the secretion of inflammatory factors, and ultimately alleviates tumor growth in ICC. The inhibition of genes related to purine metabolism can serve as basis for the study of the disease and development of treatments for ICC.

## 2. Materials and Methods

### 2.1. Clinical Sample Collection

Serum samples from five patients with ICC were collected at the Hunan Provincial People's Hospital. Patients who met the following criteria were included in this study: (1) primary resectable ICC, fine needle biopsy pathology-confirmed ICC; (2) no preoperative anticancer treatments; (3) no history of other malignancies; (4) no macroscopic invasion to the portal vein or metastasis to distant sites. Five healthy human serum samples were collected. The details of the samples are shown in Table 1. The study was approved by the ethics association of Hunan Provincial People's Hospital and the concerned hospital.

### 2.2. Cell Culture

Normal fibroblasts (NFs) (BNCC311796, BNCC, China), the human cholangiocarcinoma cell line HCCC-9810 (BNCC351917, BNCC, China), and isolated human ICC-associated fibroblasts (iCAF) were isolated in vitro after treatment with 10% fetal bovine serum (FBS) in RPMI 1640 medium (Gibco, Thermo Fisher Scientific, Inc.) and maintained at 37°C in a humidified incubator containing 5% CO_2_. In cell grouping, NFs, cocultured with HCCC-9810, was used as the NF group. The iCAF group was cocultured with iCAF and HCCC-9810 was iCAF group. When iCAF and HCCC-9810 cells were cocultured with 10 *μ*M MPA for 72 h, the iCAF + MPA group was added. The iCAF + MPA + guanosine group was incubated with 10 *μ*M MPA and 50 *μ*M guanosine when cocultured with HCCC-9810 cells for 72 h. PPAT and IMPDH1genes were silenced in HCCC-9810 cells using Nucleofector II. The control, PPAT, and IMPDH1 siRNA lentiviruses were purchased from HonorGene. The siRNA sequences are listed in Table 2.

### 2.3. ICC Datasets and Preprocessing

In total, 44 samples were collected from TCGA-CHOL (https://portal.gdc.cancer.gov/), including 36 tumor and nine normal samples. RNA sequencing (RNA-seq) data were downloaded from TCGA data portal. The fragment number per million fragments (FPKM) values was then converted to transcriptional/per million-word node (TPM) values. The microarray dataset GSE72094 was downloaded from the Gene Expression Omnibus (GEO, https://www.ncbi.nlm.nih.gov/geo/). All data were analyzed using R software (version 3.6.1) and R Bioconductor package. Eleven PM-related genes were selected to create heat maps, and box plots were constructed from the expression data of the 11 genes. The genes were subjected to survival analysis and clinical (pathologic-T, stage, grade, and sex) correlations.

### 2.4. Principal Component Analysis (PCA)

In the model calculation, we first find a straight line so that the residual sum of squares of all samples from this line is the smallest, the vector sum of squares projected in the direction of this number axis is the largest, and the direction of this line also reflects the maximum difference between samples; thus, the first principal component (PC1) is obtained. The second principal component (PC2) was obtained by finding the second line with the most significant difference along the vertical direction of the previous principal component line. The main parameter used to judge the quality of the PCA model was R2X, which represented the interpretation rate of the original data after dimensionality reduction. The closer the value is to one, the better it is. It is generally believed that R2X greater than 0.5 indicates a better model effect.

### 2.5. Partial Least-Squares Discriminant Analysis (PLS-DA)

PLS-DA is a discriminant analysis method based on the classical partial least-squares regression model. For PLS-DA, samples must be specified and grouped during analysis, after which the model automatically adds an implicit dataset *Y*. This method of model calculation forces each group to be classified, which is conducive to identifying similarities and differences between different groups.

### 2.6. Orthogonal Partial Least-Squares Discriminant Analysis (OPLS-DA)

OPLS-DA is a combination of orthogonal signal correction (OSC) and PLS-DA. Variance factors, irrelevant or orthogonal to the *Y* variable in the *X* data variable, are removed. OPLS-DA divides the differences in data table *X* into two parts according to the differences in data table *Y*. The first part represents the differences related to *Y* and the second part represents the differences unrelated to *Y* (orthogonal and vertical). OPLS-DA can distinguish between these two parts.

### 2.7. Liquid Chromatography-Tandem Mass Spectrometry (LC-MS/MS)

To perform LC-MS/MS, we first extracted metabolites from the serum. The supernatant was centrifuged at 1150*g* for 10 min at 4°C. The supernatant (100 *μ*L) was placed in a labeled tube and stored at −80°C for metabolomic analysis. Methanol (M116118-500 ml; Beijing, China) was purchased from Aladdin. Each half of the sample was separated with 300 *μ*L of cold acetonitrile, mixed, and spun for 30 s prior to analysis. Then, the mixture was centrifuged at 4°C for deproteinization, and 1 *μ*L of supernatant was injected into an ultraperformance liquid chromatography system. The NOESY-PR-1D pulse sequence was used for the detection.

### 2.8. Enzyme-Linked Immunosorbent Assay (ELISA)

ELISA kits for IL-6 (T30037024, CSB-E04638h), VEGF (E08038786, CSB-E11718h), VCAM-1 (B06038703, CSB-E04753h), OPN (V06033701, CSB-E08392h), and IL-8 (Y26037549, CSB-E04641h) were purchased. Separate wells were set up for testing the standards and samples. Each well was spiked with 100 *μ*L of the standard or sample to be tested. 100 *μ*L of biotin-labeled antibody working solution was added to each well. The plate is then overlaid with a new patch and incubated at 37°C for 1 hour. After washing, 100 *μ*L of horseradish peroxidase-labeled affinity working solution was added to each well, covered with a new plate patch and incubated at 37°C for 1 hour. The samples were then covered with a plate sticker. Each well was sequentially spiked with 90 *μ*L of substrate solution, and the reaction was developed for 15–30 min at 37°C in the dark. The optical density (OD) of each well was measured sequentially at 450 nm using an enzyme marker within 5 min of reaction termination.

### 2.9. Quantitative Real-Time PCR (qRT-PCR)

Total RNA was extracted from HCCC-9810 cells using TRIzol (15596026, Thermo, USA). The sample RNA was reverse-transcribed to cDNA according to the instructions of the Reverse Transcription Kit (CW2569, Kangwei Century, China) in a total of 40 cycles. The reaction system is 20 *μ*L. Fluorescence quantitative PCR was performed in a fluorescence quantitative RCP instrument (QuantStudio1, Thermo, USA). Reaction conditions are as follows: 40 cycles of predenaturation at 95°C for 10 min, denaturation at 94°C for 15 s, and annealing at 60°C for 30 s. The internal reference primer was *β*-actin, and the primer sequences are shown in Table 3. Using 2 *μ*g of total cDNA as a template, the relative transcription level of the target gene was calculated using the relative quantitative method (2^−△△Ct^): △ CT = △ CT experimental group − △ CT control group, △ CT = CT (target gene) − CT (ACTINB).

### 2.10. Western Blot

Total cellular protein was extracted using the total protein extraction solution RIPA kit (R0010; Solarbio, China). The protein concentration was determined according to the instructions of BCA protein quantitative kit. The protein concentration was calculated according to the standard curve. The protein was separated by 10% SDS-PAGE electrophoresis. Proteins are electrostatically transferred to the NC membrane. Substrate chromogenesis or radioautography was used to detect the protein component expressed by a specific target gene, separated by electrophoresis. The primary antibodies that were used were rabbit anti-N-cadherin (1: 5000, 22018-1-AP; Proteintech), rabbit anti-E-cadherin (1: 50000, 20874-1-AP; Proteintech), rabbit antivimentin (1: 5000, 10366-1-AP; Proteintech), rabbit anti-SOX2 (1: 600, 11064-1-AP; Proteintech), rabbit anti-CD133 (1: 800, 18495-1-AP; Proteintech), rabbit anti-PPAT (1: 1000, 15401-1-AP; Proteintech), and rabbit anti-IMPDH1 (1: 5000, 22092-1-AP; Proteintech). This was followed by incubation with horseradish peroxidase-conjugated goat anti-rabbit IgG (1: 5000, SA00001-2; Proteintech). The membrane was immersed in SuperECL Plus (K-12045-D50, Advansta, USA) for luminescence visualization using *β*-actin as an internal reference.

### 2.11. Flow Cytometry

The cells were digested and collected by trypsin without EDTA. The cells were washed with PBS (SH30256.01, Hyclone, China) twice, centrifuged at 2000 rpm for 5 min each time, and about 5 × 10^5^ cells were collected. 500 *μ*L of binding buffer was added to suspension cells. Then, 5 *μ*L of Annexin V-FITC (KGA108, KeyGEN, China) was added and mixed, followed by 5 *μ*L of propidium iodide. Within 1 hour, flow cytometry (A00-1-1102; Beckman; USA) was utilized to observe and detect.

### 2.12. Cell Counting Kit-8 (CCK-8)

Cell proliferation capacity was measured using a CCK-8 kit (NU679, Dojindo, Japan). Logarithmic growth phase cells were inoculated in 96-well plates at 5 × 10^3^ cells/well and cultured for 3 days. Cells were digested with trypsin digestion solution (C0201, Beyotime, China) to prepare cell suspensions and inoculated into 96-well plates at 1 × 10^4^/100 *μ*L. The medium was aspirated, discarded, replaced with 110 *μ*L of CCK-8 working solution (HonorGene, China), incubated for 4 h at 37°C in a 5% CO_2_ incubator, and the absorbance at 450 nm was measured using an enzyme marker (MB-530, HEALES, China).

### 2.13. Transwell

*Preparation of Cell Suspension*. The intervened cells were digested with 0.25% trypsin digestion solution, the cell suspension was prepared with serum-free basal medium, and the cell density was adjusted to approximately 1 × 10^5^/mL. The culture solution in the small chamber was discarded, and the cells were washed twice with PBS. The cells in the upper chamber were wiped with wet cotton swabs, fixed in acetone: methanol (1 : 1) for 20 min, washed twice with PBS, stained with 0.5% crystal violet for 5 min, and washed more than three times with water. The cells were observed under an inverted microscope and photographed.

### 2.14. Colony Formation Assay

*Preparation of Cell Suspension*. After 24 h of intervention, the cultured cells were digested with 0.25% trypsin digestion solution, the cell suspension was prepared with serum-free basal medium, and the cell density was adjusted to approximately 1 × 10^5^/mL. *Inoculation of Cells*. 1000 cells/2 mL of each group were inoculated in a 6-well plate, shaken to disperse, and incubated at 37°C in an incubator with 5% CO_2_. The cells were incubated at 37°C and 5% CO_2_, and the solution was changed every 2-3 d. *Staining.* The culture medium was discarded and the cells were washed twice with PBS. The washed cells were fixed with 4% paraformaldehyde (P0099, Beyotime, China) for 30 min, washed twice with PBS, stained for 5 min with 0.5% crystal violet (C0121, Beyotime, China), and washed with aseptic water more than three times. Each well was photographed and the number of clones in each well was counted.

### 2.15. Tumor Xenograft in Nude Mice

A total of 1 × 10^7^HCCC-9810 cells were suspended in 100 *μ*L of serum-free medium and mixed with equal amounts of Matrigel (Corning, USA), then injected subcutaneously into the dorsal flanks of 12 BALB/c nude mice (Hunan Slaughter Jingda Biological Co., Ltd.) at 4 weeks of age. One week after modeling, six of the mice received 120 mg/kg Mycophenolate Mofetil (MMF) orally, twice daily for 3 weeks. The control group received equal volume of excipient control. Tumor volumes were measured every 2 days with calipers and calculated using the following formula: *V*=*L* × *W*^2^/2, where *L* represents tumor length and *W* represents tumor width.

### 2.16. Hematoxylin-Eosin (HE) Staining

Sections of nude mouse tumor tissue were removed and dewaxed to water. The sections were first placed in xylene for 10 min, 2 times. The sections were then sequentially placed in 100%, 100%, 95%, 85%, and 75% ethanol for 5 min at each level, then washed with distilled water for 5 min. Stained with hematoxylin (AWI0009a, Wellbio, China) for 3 min, the sections were washed with running water for 3 s, differentiated with 1% hydrochloric acid ethanol for 3 s, and stained with 5% eosin solution for 3 min. Subsequently, the sections were dehydrated, permeabilized, sealed, and observed under a microscope (Motic, China).

### 2.17. Immunohistochemistry (IHC)

Nude mouse tumor tissue sections were immersed in 0.01 M citrate buffer (pH 6.0), heated to boiling in an electric oven or microwave oven and then disconnected, cooked continuously for 20 min, cooled for 20 min, and then removed and cooled to room temperature. We added appropriate dilutions of primary antibodies (Caspase 3 (1: 200, 19677-1-AP, PTG), Ki67 (1: 200, ab16667, Abcam), N-cadherin (1: 200, 22018-1-AP, PTG), vimentin (1: 200, 10366-1-AP, PTG), E-cadherin (1: 200, 20874-1-AP, PTG)) to the sections dropwise overnight at 4°C. The sections were incubated with rabbit-IgGantibody-HRP multimers dropwise for 30 min at 37°C. The sections were then incubated for 1–5 min at room temperature after the addition of 50–100 *μ*L of premade chromogenic DAB working solution dropwise. We used hematoxylin to restain the sections for 5–10 min. Finally, the staining results were observed by microscopy.

### 2.18. Immunofluorescence (IF)

The sections were placed in three xylene solutions for 20 min each time. They were then treated with 100%, 95%, 85%, and 75% ethanol for 5 min each. The sections were then soaked in distilled water for 5 min and then placed in ethylene diamine tetraacetic acid (EDTA) solution (pH 9.0), boiled by continuous microwaving for 24 min, and cooled to room temperature. Each section was then placed in sodium borohydride solution at room temperature for 30 min and rinsed with water for 5 min. This was followed by exposure to Sudan black dye solution at room temperature for 5 min and rinsing with water for 3 min. Appropriate dilution of primary antibody CD133 (1: 50, ab1989, Abcam) and SOX2 (1: 50, 67793-1-IG, Proteintech) were added overnight at 4°C. Secondary antibody: 50–100 *μ*L anti-Rabbit + IgG (*H* + *L*) (1: 5000, SA00013-2, Proteintech) fluorescent antibody was added and incubated at 37°C for 90 min.

### 2.19. Statistical Analyses

The measurement data are expressed as mean ± standard deviation (SD). The count information was presented as a percentage. Each test was repeated independently three times. All data were analyzed by using GraphPad Prism 8.0 software (La Jolla, CA, USA). Kolmogorov−Smirnov test and exploratory descriptive statistics test were used to analyze whether the data conformed to a normal distribution and homogeneity of variance. The measurement data obeyed the normal distribution and homogeneity of variance. The data were analyzed by the parametric test. The unpaired Student's *t*-test was used to compare the data of two groups that were not one-to-one correspondence. One-way ANOVA and Tukey's posthoc test were used to compare data among three groups. The degrees of freedom, the sum of squares, and the mean of the sum of squares were used to present the analysis results. The difference was statistically significant at *P* < 0.05.

## 3. Results

### 3.1. Detection of Differential Metabolic Small Molecules in Serum by Metabonomics

**Figure 1:**
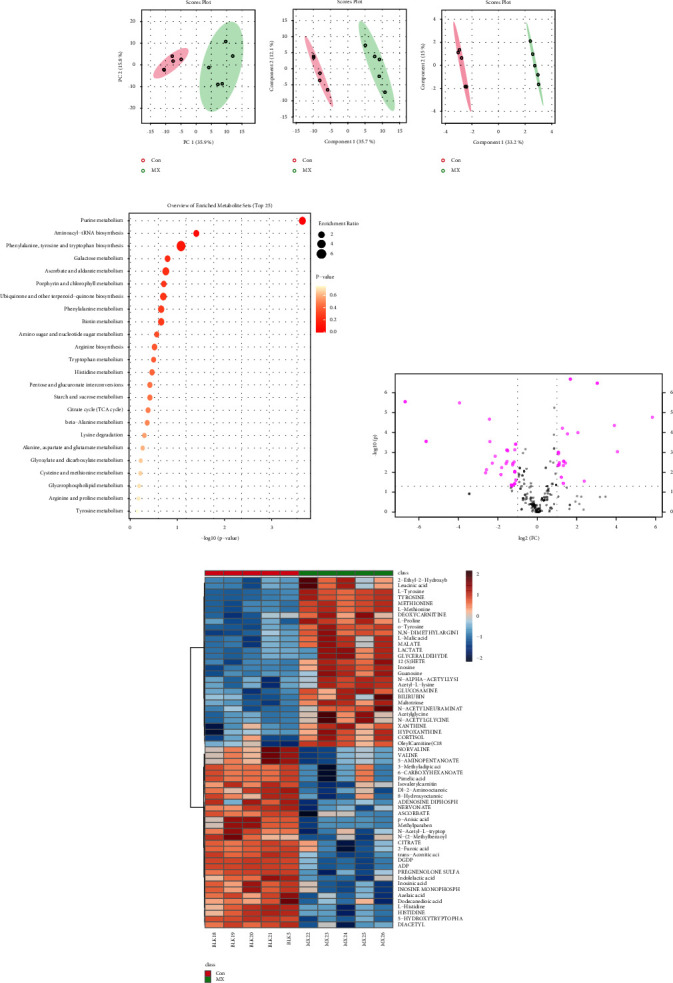
Serum metabolites. (a) PCA analysis. (b) PLS-DA analysis. (c) OPLS-DA analysis. (d) The top 25 metabolites were enriched. (e) Differential metabolite of volcano plot (|log2 (FC)| > 1). (f) The top 60 differential metabolites were screened.

In order to investigate whether the metabolism of patients with ICC was abnormal, serum samples from clinical ICC patients and healthy persons were collected for metabolic analysis. Principal component analysis (PCA) (Figure 1(a)), partial least-squares discriminant analysis (PLS-DA) (Figure 1(b)), and orthogonal partial least-squares discriminant analysis (OPLS-DA) (Figure 1(c)) showed significant differences in metabolites between patients with ICC and who were healthy. Serum purine metabolism, aminoacyl-tRNA biosynthesis, and phenylalanine metabolism were significantly enriched in patients with ICC (Figure 1(d)). Volcano plots were used to show differential metabolites at high and low levels (Figure 1(e)). The results of Figure 1(f) show that purine metabolism metabolites such as inosine, guanosine, hypoxanthine, and xanthine were increased in ICC patients' serum, and inosine monophosphate, adenosine diphosphate ribose, ADP, and DGDP were significantly decreased. L-Methionine, L-Tyrosine, and L-Proline were increased and L-Histidine was decreased in aminoacyl-tRNA biosynthesis. Among them, L-Tyrosine is also one of the important products of the metabolic pathway of phenylalanine metabolism. Therefore, the differential purine metabolism may have a regulatory role in the development of ICC.

### 3.2. Purine Metabolism Genes Are Upregulated in ICC

**Figure 2:**
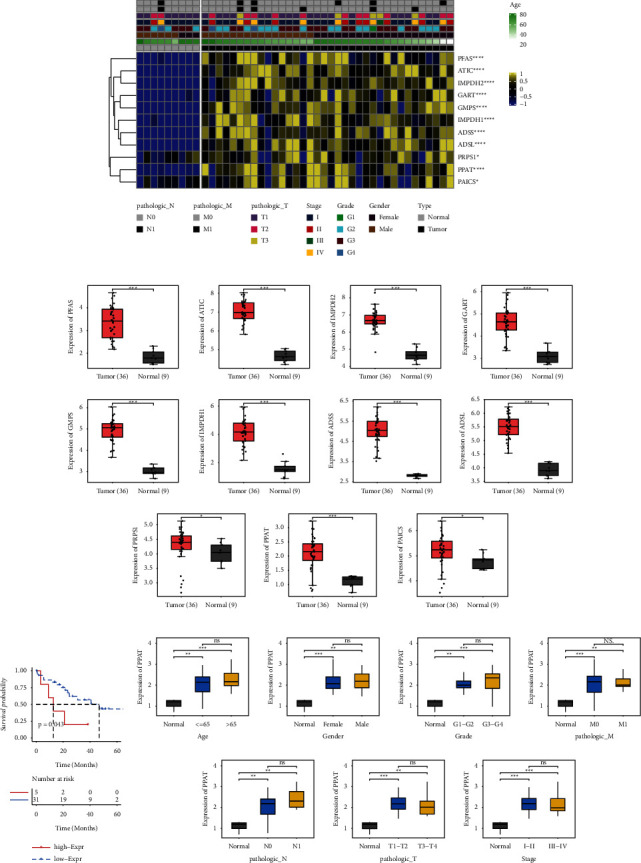
Purine metabolism gene. (a) Expression levels of purine metabolism-related genes in ICC based on age, gender, tumor stage, and neoplasm histologic grade. (b) Expression of purine metabolism-related genes in ICC. (c) Kaplan–Meier survival curve based on PPAT gene. (d) PPAT gene expression was analyzed based on clinical indicators. ^*∗*^*P* < 0.05. ^*∗∗*^*P* < 0.01. ^*∗∗∗*^*P* < 0.001. ns, *P* > 0.05.

To further observe the effect of purine metabolism in ICC, we constructed a clinical model of purine metabolism-related genes and ICC (Figure 2(a)). Compared with the normal group, the tumor group showed higher expression of purine metabolism-related genes such as PFAS, ATIC, IMPDH2, GART, GMPS, IMPDH1, ADSS, ADSL, PRPS1, PPAT, and PAICS (Figure 2(b)). Next, according to the 11 genes survival curve analysis, only the high and low expression of PPAT had survival differences in ICC (Figure 2(c)). The expression of PPAT was significantly higher in the tumor group compared to the normal group, which was statistically significant. In ICC patients, PPAT expression differed less among age, sex, grade, pathologic_M, pathologic_N, pathologic_T, and stage (Figure 2(d)). It suggested that PPAT may be an important gene affecting ICC.

### 3.3. Downregulation of Purine Metabolism Gene PPAT Could Limit Cell Growth and Metastasis of HCCC-9810

**Figure 3:**
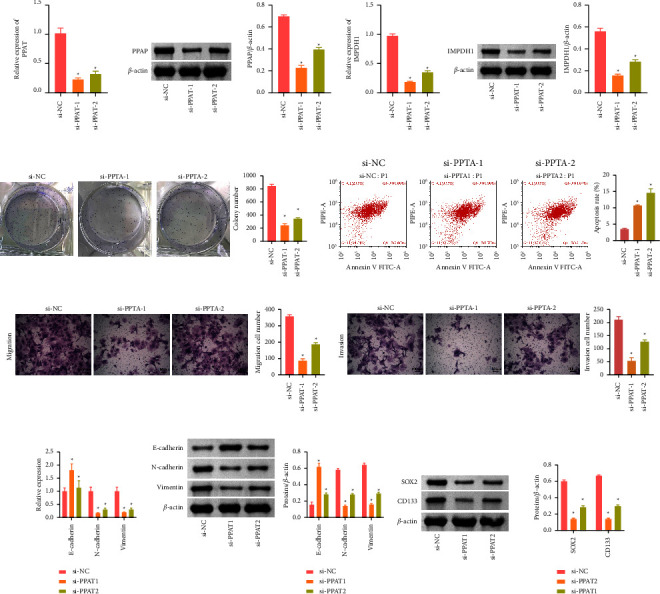
The control purine metabolism-related gene PPAT can affect HCCC-9810. (a) qRT-PCR and Western blot were used to detect the expression of PPAT. (b) qRT-PCR and Western blot were used to measure the expression of IMPDH1. (c) Colony formation assay was performed to test the cloning ability of HCCC-9810. (d) Apoptosis rate of HCCC-9810 was analyzed by flow cytometry. (e) Transwell was used to detect migration of HCCC-9810. (f) HCCC-9810 invasion was detected by transwell. (g) Western blot was used to detect epithelial-mesenchymal transformation (EMT) markers of E-cadherin, N-cadherin, and vimentin. (h) The tumor stem cell markers CD133 and SOX2. ^*∗*^*P* < 0.05 compared with the si-NC group. Data among multiple groups were analyzed by one-way ANOVA, followed by Tukey's post hoc test.

To investigate the effect of PPAT on ICC, we performed in vitro experiments to observe the effect of PPAT on HCCC-9810. Firstly, a PPAT siRNA cell model was constructed. The level of PPAT decreases significantly, indicating that the cellular silencing PPAT model was successfully constructed (Figure 3(a)). The level of IMPDH1 decreased in the presence of PPAT inhibition, which implied a regulatory relationship between IMPDH1 and PPAT (Figure 3(b)). We further examined the cell function situation. Compared with the si-NC group, the number of colonies (Figure 3(c)) and the number of migration and invasion cells (Figures 3(e) and 3(f)) decreased and the apoptosis rate increased in the si-PPAT1 and si-PPAT2 groups (Figure 3(d)). The level of protein of N-cadherin, vimentin, SOX2, and CD133 decreased and the E-cadherin increased in the si-PPAT1 and si-PPAT2 groups (Figures 3(g) and 3(h)). In conclusion, PPAT was able to inhibit the cell growth and migration of HCCC-9810.

### 3.4. IMPDH1 Could Regulate HCCC-9810 Cells

**Figure 4:**
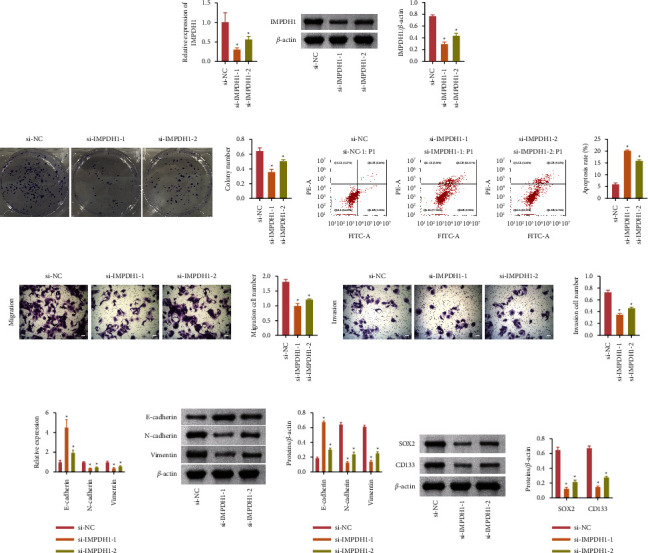
IMPDH1 was able to inhibit the cell growth and migration of HCCC-9810. (a and b). The level of mRNA and protein of IMPDH1. (c) The cloning ability of HCCC-9810. (d) The apoptosis rate of HCCC-9810 analyzed by flow cytometry. (e) Migration of cell number of HCCC-9810. (f) HCCC-9810 invasion was detected by transwell. (g) Western blot was used to detect E-cadherin, N-cadherin, and vimentin. (h) The level of protein of cell markers CD133 and SOX2. ^*∗*^*P* < 0.05 compared with the si-NC group. The data are expressed as mean ± SD. Comparisons among multiple groups were analyzed using one-way ANOVA.

The abovementioned results suggested that PPAT has an inhibitory effect on IMPDH1, which inhibited the cellular function of HCCC-9810. We further explored whether IMPDH1 could affect HCCC-9810 cells directly. An IMPDH1 siRNA cell model was constructed. The level of IMPDH1 decreased significantly, indicating that the cellular silencing IMPDH1 model was successfully constructed (Figures 4(a) and 4(b)). Compared with the si-NC group, the number of colonies (Figure 4(c)) and the number of migration and invasion cells (Figures 4(e) and 4(f)) decreased and the apoptosis rate increased in the si-IMPDH1-1 and si-IMPDH1-2 groups (Figure 4(d)). The level of protein of N-cadherin, vimentin, SOX2, and CD133 decreased and the E-cadherin increased in the si- IMPDH1-1 and si- IMPDH1-2 groups (Figures 4(g) and 4(h)). In conclusion, IMPDH1 was able to inhibit the cell growth and migration of HCCC-9810.

### 3.5. Purine Metabolism Could Mediate iCAF to Regulate HCCC-9810 Cells

**Figure 5:**
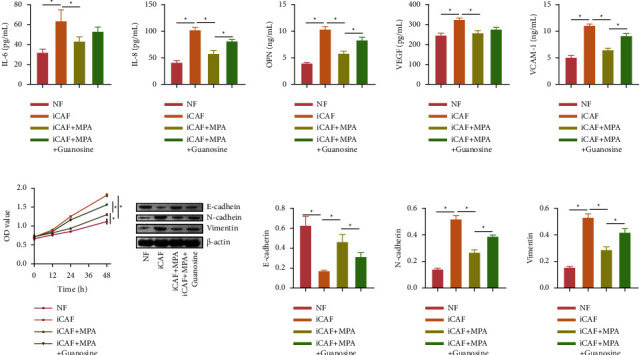
Changes in purine metabolism may influence the cellular biological behavior of ICC-associated fibroblasts. (a) Cytokine secretion of supernatant was detected by ELISA: IL-6, IL-8, OPN, VEGF, and VCAM-1. (b) CCK-8 was used to detect cell proliferation. (c) Western blot was used to detect epithelial-mesenchymal transformation (EMT) markers. ^*∗*^*P* < 0.05. The measurement data are expressed as mean ± SD. Data among multiple groups were analyzed by one-way ANOVA, followed by Tukey's post hoc test.

The abovementioned results suggested that PPAT has an inhibitory effect on IMPDH1, which inhibited the cellular function of HCCC-9810. We further explored whether MPA, an inhibitor of IMPDH1, and guanosine, a purine metabolite, could affect iCAF to regulate HCCC-9810 cells. First, we characterized NF cells and iCAF with morphological and iCAF cellular markers *α*-SMA (84.87%), CD34 (0.02%), and CD45 (0.1%) results to determine that the cells could be used for subsequent experiments (Figure S1). Compared with the NF group, HCCC-9810 cells in the iCAF group showed increased secretion of inflammatory factors IL-6, IL-8, OPN, VEGF, and VCAM-1 (Figure 5(a)) and rapid cell proliferation (Figure 5(b)), which indicated that HCCC-9810 cells tended to become malignant under the influence of iCAF cells. Furthermore, western blot was used to detect epithelial-mesenchymal transition EMT indicators, and the data results showed that N-cadherin and vimentin expression increased and E-cadherin expression was inhibited (Figure 5(c)). ICAF promoted EMT progression in HCCC-9810 cells. However, MPA drug was able to inhibit the secretion of inflammatory factors IL-6, IL-8, OPN, VEGF, and VCAM-1 in HCCC-9810 cells in the context of their tendency to become malignant. MPA reduced the growth of HCCC-9810 cells. After the intervention of adding the metabolite guanosine, the secretion of inflammatory factors IL-6, IL-8, OPN, VEGF, and VCAM-1 increased in HCCC-9810 cells, cell proliferation was accelerated, N-cadherin and vimentin expression increased, and E-cadherin expression was inhibited. This shows that MPA can alleviate the secretion of inflammatory factors by HCCC-9810 cells, while the purine metabolite guanosine can reverse the effect of MPA.

### 3.6. Inhibition of Purine Metabolism Gene IMPDH1 Inhibited the Development of ICC in Mice

**Figure 6:**
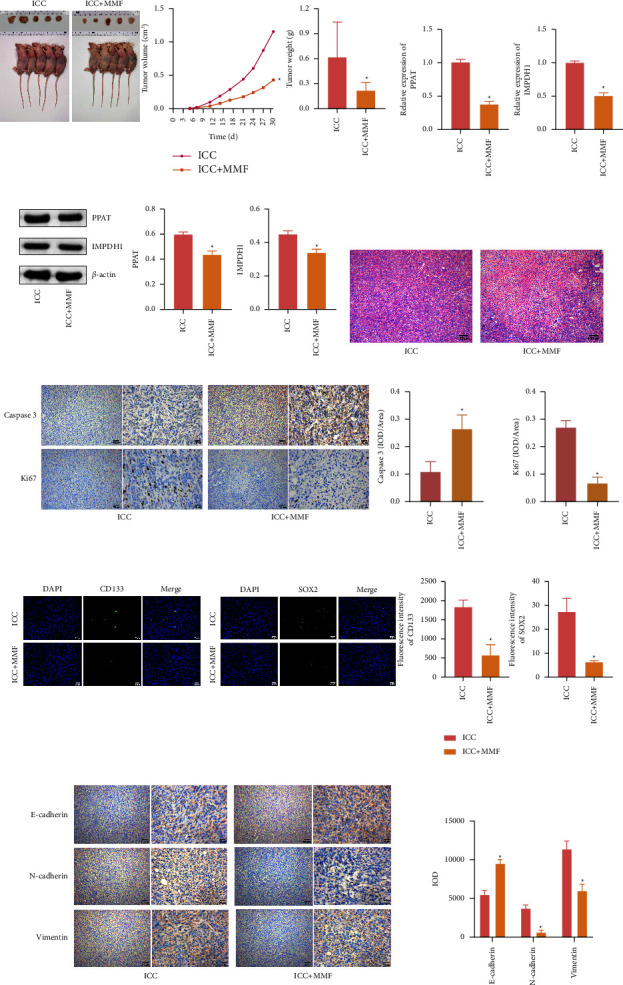
MMF inhibited the development of ICC in mice. (a) Tumor volume and weight statistics. (b and c) qRT-PCR and western blot were used to detect mRNA and protein levels of PPAT and IMPDH1 genes. (d) HE was used to measure tumor pathology in each group. (e) IHC was used to detect the positive rate of proliferation (Ki67) and apoptosis (caspase 3) genes in tissue. (f) IF was used to detect stem cell characteristics (CD133 and SOX2) markers in tissue. (g) IHC was used to measure the positive rate of E-cadherin, N-cadherin, and vimentin in tissue. ^*∗*^*P* < 0.05 compared with the ICC group. The measurement data are expressed as mean ± SD. The unpaired *t*-test was used to analyze comparisons between two groups. *n* = 6.

The abovementioned results indicated that MPA could inhibit the proliferation of HCCC-9810 in vitro, and we further investigated the effect of MMF, a prodrug of MPA, in ICC rats in vivo. We constructed a nude mouse model of ICC, and the tumor volume and tumor weight of nude mice were significantly reduced after the addition of MMF (Figure 6(a)). Compared with the ICC group, the expression levels of PPAT and IMPDH1 were decreased in the ICC + MMF group (Figures 6(b) and 6(c)). The next observation of tumor tissue morphology showed that a large number of vacuoles appeared in the tumor tissues after drug administration, probably due to more cell death in the tumor tissues (Figure 6(d)). The positivity of apoptosis-associated gene Caspase 3 and proliferation-associated gene Ki67 in tumor tissues was further examined. The data results showed that Caspase 3 expression was elevated and Ki67 expression was inhibited by the intervention of MMF. This indicates that MMF can promote the initiation of tumor cell death program (Figure 6(e)). The tumor stem cell markers CD133 and SOX2 were reduced by MMF intervention (Figure 6(f)). IHC was used to detect epithelial-mesenchymal transition EMT indicators, and the data results showed that N-cadherin and vimentin expression inhibited and E-cadherin expression was increased by MMF intervention (Figure 6(g)). In summary, inhibition of the purine metabolism gene IMPDH1 inhibited the growth of tumor in mice.

## 4. Discussion

In this study, PCA showed statistically significant differences in metabolites between the ICC and control samples. Interestingly, 60 differential metabolites were found in the samples obtained from ICC patients compared to those in the control group. Notably, guanosine, a purine metabolite, was found to be harmful to the prevention and treatment of ICC. In this study, R-linguistic analysis was used to establish a prognostic model for ICC, and the purine metabolism-related genes PPAT and IMPDH1 were screened. In vitro and in vivo experiments verified that inhibition of PPAT and IMPDH1 could inhibit the progression of ICC.

Considering the diagnostic uncertainty of most ICC cases, metabolomic approaches may not only elucidate the disease pathogenesis of ICC but may also provide information for diagnosis, prediction, or prognosis [19]. Guanine nucleotides are building blocks of DNA and RNA and are also used by the guanine binding protein family (g-proteins) for a large number of cellular functions, such as cytoskeletal rearrangement, membrane transport, protein synthesis, and signal transduction [20]. The biosynthetic pathway of GTP is important for the progression of many tumors, such as glioblastoma [21], small cell lung cancer subgroup [22], solid and serous malignancies, and clear cell renal cell carcinoma [23]. GTP is a well-known biological metabolite in the context of cancer. The biosynthesis of GTP and its intracellular levels are well-coordinated and regulated, at least in part, by IMPDH1. In in vitro experiments, exogenous guanosine effectively promoted the expression of PPAT and IMPDH1 in HCCC-9810 cells and stimulated the secretion of the inflammatory factors IL-6, IL-8, OPN, VEGF, and VCAM-1. Under PPAT silencing, the expression of IMPDH1 was subsequently reduced, and this finding suggests that there may be an interaction between IMPDH1 and PPAT. We will consider further studies on the regulatory mechanisms between PPAT and IMPDH1.

As for the role of IMDPH in tumors, recent studies have shown that high IMPDH2 expression was associated with cancer progression [24]. Tumors from mice and humans express high levels of IMPDH1 and IMPDH2, the rate-limiting enzymes for de novo guanine nucleotide synthesis [25]. The gene encoding these enzymes was MYC. Drug inhibition of IMPDH1 can reduce the expression of RNA polymerase I-dependent ribosomal RNA and effectively inhibit the growth of lung cancer cells in vitro [26]. According to recent findings, including the regulation of IMPDH1 through cell cytosolic assembly, metastable inhibition of these enzymes is possible and key differences exist between IMPDH1 isoforms [17]. Specially designed molecules may be developed to target IMPDH1 with minimal immunosuppressive effects but maximal tumorigenic inhibition, possibly by modulating the aggregation of specific IMPDH1 isoforms or drugs with different tissue penetration capabilities [27]. We used MPA as an inhibitor of IMPDH1 in vitro and found that HCCC-9810 cell growth, migration, and invasion were significantly inhibited.

As cancer therapy is moving toward personalization, one approach is to use anticancer drugs encapsulated in nanoparticles, such as liposomes or nanopolymers, and these are taken up by ICC via endocytosis, leading to higher intracellular concentrations and enhanced anticancer drug efficacy [28]. Arsenate plasmonic complex-based chemotherapy is paving the way for efficient nanomedicine-enabled boronate affinity-based arsenic chemotherapeutics for on demand site-specific cancer combination treatment of glioblastoma [29]. The application of nanomedicine is also expected to bring new hope to liver cancer [30] and pancreatic ductal adenocarcinoma treatment [31, 32]. In addition, several targeting strategies have been proposed to deliver drugs specifically to ICC. For this purpose, bile acid derivatives have been used as “Trojan horses” to enhance the uptake by cancer cells of antitumor fractions in enterohepatic circulation, such as cisplatin, which are chemically bound to bile acids and are recognized and transported through the plasma [33, 34]. We used oral MMF in in vivo experiments in nude mice with tumorigenesis, where tumor volume and weight were reduced, tumor tissue cell growth was inhibited, and cancer cell stemness was reduced. We were unable to conduct clinical trials owing to funding and conditions. In future, we will try to overcome this limitation and perform more in-depth studies.

In conclusion, inhibition of the production of the purine metabolite guanosine inhibits the development of ICC. In vitro, we verified that the purine metabolism-related genes PPAT and IMPDH1 were highly expressed in HCCC-9810 cells. Silencing PPAT and IMPDH1 effectively inhibited the malignant progression of HCCC-9810 cells. In vivo, MMF was shown to inhibit IMPDH1 expression, inhibit tumor growth, and promote apoptosis of HCCC-9810 cells. Silencing the purine metabolism gene IMPDH1 has potential value in the treatment of ICC.

## Figures and Tables

**Table 1:** Baseline characteristics of intrahepatic cholangiocarcinoma (ICC) patients and normal.

Variables	ICC (*n* = 5)	Normal (*n* = 5)	*P* value
Age^*∗*^ (years)	62 (54–67)	58.0 (50–64)	0.421
Gender (male/female)	2/3 (40.0/60.0)	2/3 (40.0/60.0)	1.000
HBsAg (negative/positive)	4/1 (80.0/20.0)	0/5 (0.0/100.0)	0.010
Cirrhosis (no/yes)	3/2 (60.0/20.0)	0/5 (0.0/100.0)	0.114
WBC^*∗*^ (10^9^/L)	6.3 (3.5–9.5)	6.1 (4.1–8.6)	0.316
Hemoglobin^*∗*^ (g/dL)	14.6 (11.2–19.1)	14.8 (12.8–19.8)	0.150
PLT^*∗*^ (10^9^/L)	194 (100–436)	178 (120–372)	0.285
PT^*∗*^ (seconds)	12.2 (9.5–17.0)	12.2 (9.5–17.0)	0.153
TBIL^*∗*^ (*μ*mol/L)	11.2 (9.2–18.5)	7.9 (7.5–17.3)	0.147
ALB^*∗*^ (g/L)	42.7 (31.8–53.1)	43.6 (38.5–54.9)	0.263
ALT^*∗*^ (U/L)	40.1 (19.8–54.6)	23.8 (8.9–49.1)	0.129
AST^*∗*^ (U/L)	35.1 (22.4–46.5)	23.0 (16.7–23.5)	0.017
CEA^*∗*^ (ng/mL)	5.3 (0.3–6.1)	1.2 (0.5–2.1)	0.046
CA199^*∗*^ (U/mL)	336.7 (30.1–2501.2)	8.6 (8.2–12.2)	0.104
Tumor size (cm)	5.5 (4.0–6.4)		
Tumor number (single/multiple)	4/1 (80.0/20.0)		

Data are *n* (%) and ranges. ^*∗*^Presented as median and ranges. ALT: alanine aminotransferase; AST: aspartate aminotransferase; ALB: albumin; CEA: carcinoembryonic antigen; CA199: carbohydrate antigen199; HBsAg: hepatitis B surface antigen; ICC: intrahepatic cholangiocarcinoma; PLT: platelet; PT: prothrombin time; WBC: white blood cell.

**Table 2:** siRNA sequences.

Sites	Sequences
Target sequence PPAT1	GAGTGCTGGTATTGTGACTAGTGAT
Target sequence PPAT2	CACACAAGGGAATGGGTCTTGTAAA
Target sequence IMPDH1-1	CAGAAGAATGGTACAAATCCAAG
Target sequence IMPDH1-2	CCCTTAAAGGAACCAATGAGTCC

**Table 3:** Primer sequences.

Gene	Gene ID	Sequences (5′-3′)
H-PRPS1	5631	F: TGAGCCGCAGTAGTTCTTCAG
		R: GGCAACAGTTCTTCAACTGGC

H-PPAT	5471	F: TGGTAGCTGGTAGGGTTGGA
		R: AGGCTAGCCAATGCTTGTGT

H-PAICS	10606	F: CAGCACCCAACAGTAGCGTA
		R: AGCGAGGCAGAACTCAAGTC

H-PFAS	5198	F: ACGATTCGAGGTGCTCTGTG
		R: CCTTGCAGTTTCCTCCGAGT

H-GAPT	202309	F: CACCCGGTGTCGGTTTCATT
		R: CCTTTCCAGGCCAGCGTATGTT

H-GMPS	8833	F: TGTCATGAAGCTTCCGTGTT
		R: TCCCCCGTTAACATTGAGCA

H-ADSS	11566	F: AAGGAGCTACCTGTCAACGC
		R: CCAACGCCAGCTGAAAACTC

H-ADSL	158	F: TAAATTCCGGACATGGCGGC
		R: CTGCAGCTTTTGGACAGCAG

H-ATIC	471	F: CGGCCAGCTCGCCTTATTTA
		R: CCAAGAGCGGTCAGGTTTCT

H-IMPDH1	3614	F: CGACACCCCGTTCAGAACTAT
		R: TCAGGTAGTCCGCCATGCTA

H-IMPDH2	3615	F: AGGCGGTATTGGCTTCATCC
		R: GCCTGTGTCTGTGATTGGGA

M-PPAT	231327	F: GAAGGAGGCAGTCAGTAGCG
		R: AGTCTCCAGAAGCGATGCAC

M-IMPDH1	23917	F: CTACGAGCCGGAGAGCATGG
		R: TGTCAGGTCCACTTCATCAGC

H-N-cadherin	35070	F: GGGAAATGGAAACTTGATGGCA
		R: TGGAAAGCTTCTCACGGCAT

H-vimentin	7431	F: CCCTTGACATTGAGATTGCCACC
		R: ACCGTCTTAATCAGAAGTGTCCT

H-E-cadherin	114424	F: ATTTTTCCCTCGACACCCGAT
		R: TCCCAGGCGTAGACCAAGA

H-ACTINB	60	F: ACCCTGAAGTACCCCATCGAG
		R: AGCACAGCCTGGATAGCAAC

M-ACTINB	11461	F: ACATCCGTAAAGACCTCTATGCC
		R: TACTCCTGCTTGCTGATCCAC

## Data Availability

All data included in this study are available from the corresponding authors upon request.

## Supplementary Materials

Supplementary Materials Figure S1: Markers of iCAF cells. (A) Morphological characteristics of NFs and morphological characteristics of iCAF cells. (B) Primary iCAF were identified by flow cytometry, which were positive for *α*-SMA and negative for CD34 and CD45.
