# Supplementary figures and images for: The Role of Purine Metabolism-Related Genes PPAT and IMPDH1 in the Carcinogenesis of Intrahepatic Cholangiocarcinoma Based on Metabonomic and Bioinformatic Analyses
Source: J Oncol. 2023 Jan 20;2023:5141836. doi: 10.1155/2023/5141836 (PMC9883099; doi:10.1155/2023/5141836)

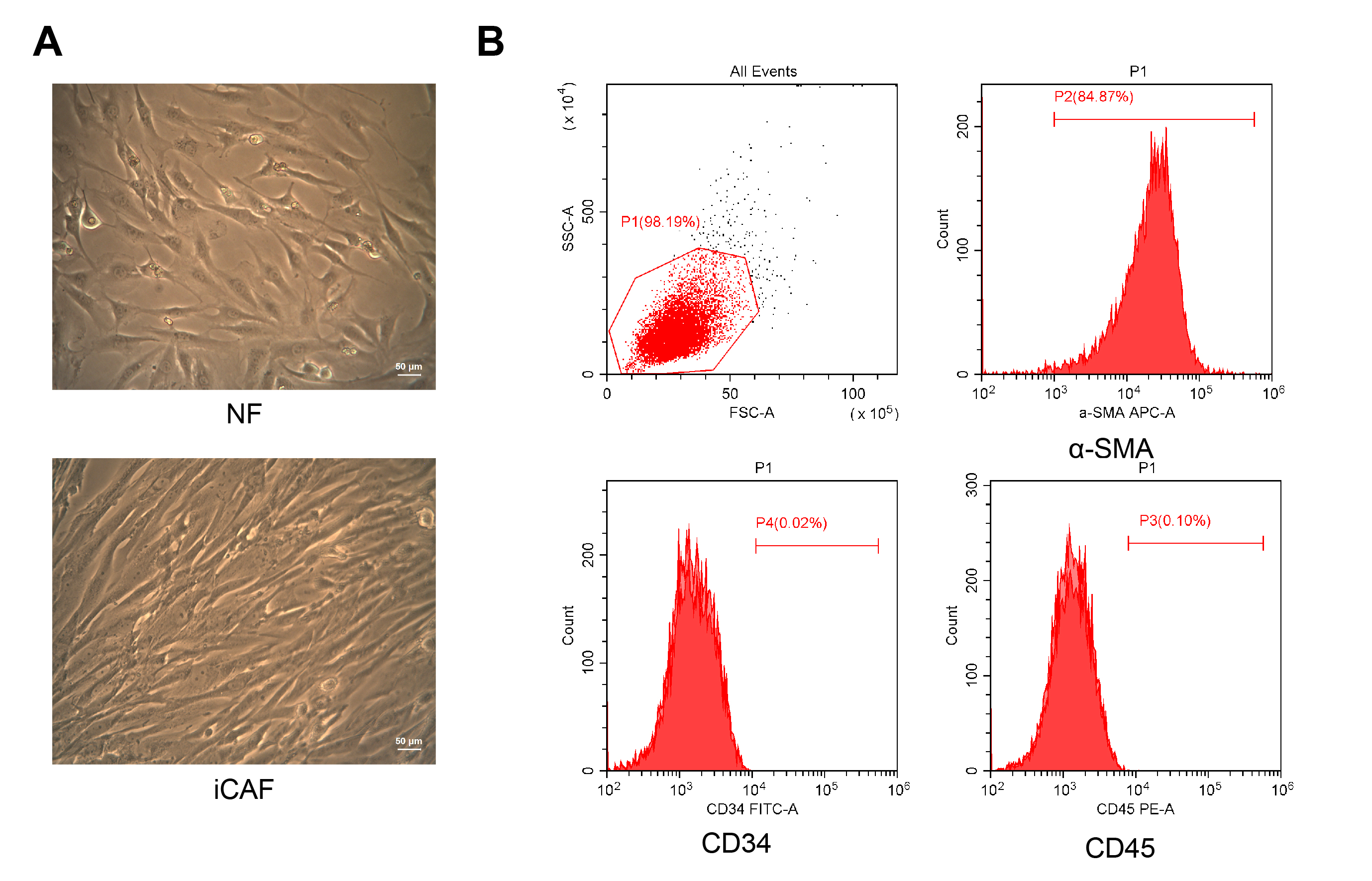

Supplement: Supplementary Materials — Figure S1: Markers of iCAF cells. (A) Morphological characteristics of NFs and morphological characteristics of iCAF cells. (B) Primary iCAF were identified by flow cytometry, which were positive for α-SMA and negative for CD34 and CD45. [file 5141836.f1.zip › Supplementary figure 1 (1).jpg]

**A**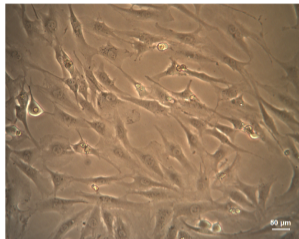**NF**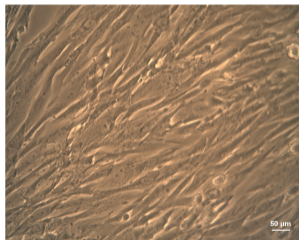**iCAF****B**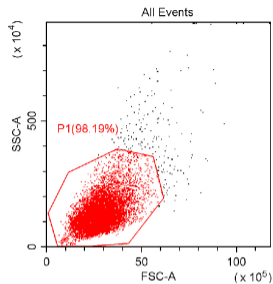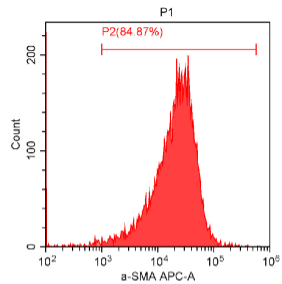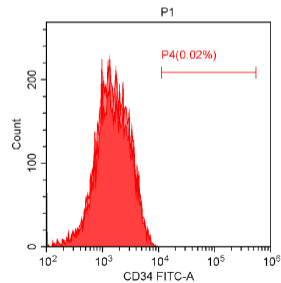**CD34**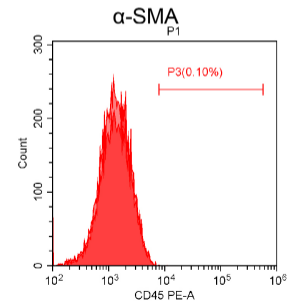**CD45**

Supplement: Supplementary Materials — Figure S1: Markers of iCAF cells. (A) Morphological characteristics of NFs and morphological characteristics of iCAF cells. (B) Primary iCAF were identified by flow cytometry, which were positive for α-SMA and negative for CD34 and CD45. [file 5141836.f1.zip › Supplementary figure 1.pdf]
